# Photo-Fenton Degradation of Pentachlorophenol: Competition between Additives and Photolysis

**DOI:** 10.3390/nano9081157

**Published:** 2019-08-13

**Authors:** Erika Pia Vergura, Sara Garcia-Ballestreros, Rosa Francisca Vercher, Lucas Santos-Juanes, Alessandra Bianco Prevot, Antonio Arques

**Affiliations:** 1 Chemistry Department, University of Torino, 10125 Torino, Italy; 2 Group of Advanced Oxidation Processes, Department of Textile and Paper Engineering, Universitat Politècnica de València, 03801 Alcoy, Spain

**Keywords:** pentachlorophenol, photo-Fenton, solar light, bio-based substances, urban waste

## Abstract

In the present work, the photo-Fenton degradation of pentachlorophenol (PCP, 1 mg/L) has been studied under simulated and natural solar irradiation; moreover, the effect on the process efficiency of urban waste-derived soluble bio-based substances (SBO), structurally comparable to humic acids, has been investigated. Experiments showed a crucial role of PCP photolysis, present in the solar pilot plant and hindered by the Pyrex^®^ filter present in the solar simulator. Indeed, the SBO screen negatively affects PCP degradation when working under natural solar light, where the photolysis of PCP is relevant. In contrast, in the absence of PCP photolysis, a significant improvement of the photo-Fenton process was observed when added to SBO. Furthermore, SBO were able to extend the application of the photo-Fenton process at circumneutral pH values, due to their ability to complex iron, avoiding its precipitation as oxides or hydroxides. This positive effect has been observed at higher concentration of Fe(II) (4 mg/L), whereas at 1 mg/L, the degradation rates of PCP were comparable in the presence and absence of SBO.

## 1. Introduction

Fenton and photo-Fenton are among the advanced oxidation processes that have gained more attention from researchers in recent years. In these processes, a mixture of catalytic iron salts and sacrificial amounts of hydrogen peroxide, are able to generate highly oxidizing species (e.g., hydroxyl radical) [1]. Although promising results have been achieved, even using sunlight as an irradiation source [2], this approach is not free of drawbacks, mainly due to the highly acidic conditions required, iron present in solution in the more active species, and to prevent iron precipitation as oxide or hydroxide that inactivate the Fenton process. Different strategies are being developed to drive photo-Fenton at a milder pH, such as the use of complexing agents [3]. In this context, some recent papers have indicated that humic substances can form photo-chemically active iron complexes at circum-neutral pH [4,5,6].

Recently, urban bio-wastes have been shown as potential cost-effective renewable source of soluble bio-based substances, including SBO, whose chemical nature was demonstrated to be very similar to natural organic matter, such as humic substances [7,8]. SBO have been produced at relatively large scales by alkaline hydrolysis of urban bio-wastes, and sampled from various process streams of the AceaPinerolese waste treatment plant, located in Piemonte, Italy. More recently, other humic-like substances (HLS) have been isolated from oil mill solid wastes [9]. These substances have been demonstrated to be photo-active and able to catalyze degradation of pollutants, namely phenols, although the process is slow [10]. On the other hand, their ability to complex iron [11], and to drive efficiently photo-Fenton to pH values slightly above 5, has been demonstrated [12,13].

However, different opposite effects can be found, which might result in an enhancement or a decrease of the pollutant degradation rate. Main effects are: (a) Generation of reactive species by the humic substances, (b) extending the efficient domain of photo-Fenton towards circumneutral pH via iron complexation, (c) a competitive/scavenging role of the humic compounds for the reactive species generated, (d) an inner filter effect, due to the light absorption by the humics that decrease the number of photons reaching the pollutant or the photocatalytic system, and (e) a surface active role that might result in an enhanced solubilization of scarcely soluble pollutants and/or a pre-association of the HLS/Fe with the pollutant. Hence, the HLS-assisted processes might be very sensitive to slight changes in the experimental conditions, as a result of a balance in the beneficial and detrimental effects.

Studying the influence of experimental conditions in the photocatalytic degradation of pollutants, the presence of humic-like substances seems to be be significant. In this work, pentachlorophenol (PCP) is the target pollutant. PCP is an organochlorine compound used as herbicide, insecticide, fungicide, algaecide, wood preservative, and as an ingredient in anti-fouling paint, although its use has declined due to its high toxicity and slow biodegradation [14,15]. Nonetheless PCP is still released into surface waters from the atmosphere by wet deposition, from soil by run-off and leaching, and from manufacturing and processing facilities. As PCP is generally used for its properties as a biocidal agent, there is considerable concern about adverse ecosystem effects in areas of PCP contamination; therefore, considerable research efforts have been devoted to the development of abiotic remediation treatment, such as the advanced oxidation processes (AOPs) to remove it completely from surfaces [16].

On this background, the present research aims to study the feasibility of photo-Fenton degradation of PCP in aqueous solution, assisted by HLS (in particular, SBO obtained from green compost). For this purpose, the photo-degradation of PCP will be studied under different conditions, tuning parameters, such as HLS or iron concentration, the H_2_O_2_ amount, or irradiation source (real and simulated).

## 2. Materials and Methods

### 2.1. Reagents

The HLS were isolated from composted urban bio-wastes (urban public park trimming and home gardening residues), aged for more than 180 days, and obtained from the ACEA Pinerolese Industriale S.p.A. waste treatment plant located in Pinerolo (Italy). This procedure has been described in detail elsewhere [8], and consists of the basic digestion of the starting material, and the isolation of the HLS, either by nano-filtration or by precipitation upon acidification of the sample. 

The composition of the HLS employed in this paper was reported in a previous paper [17]. The amount of carbon was ca. 38%, which results in 72% of volatile solids. This is close to the typical values for humic substances. The amount of aromatic groups was slightly below 10% of the total amount of carbon. The molecular weight, determined by size exclusion chromatography, was around 4500 Da, while its hydrodynamic radius was 135 nm [9].

Pentachlorophenol was purchased from Sigma-Aldrich (Saint Louis, MO, USA) and used as received. All other chemicals employed in this work were obtained from Panreac (Barcelona, Spain) and used as received. Water was Milli Q grade.

### 2.2. Reactions and Set-Up

Laboratory scale experiments were carried out in an open cylindrical Pyrex vessel (55 mm i.d.). For each experiment, the reactor was loaded with 250 mL of solution, containing PCP (1 mg/L), HLS (in the range 0–20 mg/L), iron (1–5 mg/L), and hydrogen peroxide (between 1.15 mg/L, which is the stoichiometric amount required to completely oxidize 1 mg/L of PCP, and four times this amount, namely 4.6 mg/L). The pH was adjusted to the desired value (2.8, 5 or 7) by adding diluted sulfuric acid. The samples were irradiated by means of a solar simulator (Oriel 81160), equipped with a 300 W Xenon Short Arc Lamp, where the spectrum closely matches the solar one. In some experiments, a Pyrex glass filter was employed to cut off the residual radiation emitted by the simulator in the range 280–300 nm. In order to monitor the PCP removal, the samples were periodically taken from the reaction mixture and diluted 1:1 with methanol to quench further reactions, due to the remaining H_2_O_2_; then, they were flown through polypropylene filters with pore diameter of 0.45 μm (VWR).

Sunlight irradiations were performed in a Solardetox Acadus-2001, and supplied by Ecosystem (Barcelona, Spain) [18]. Briefly, it consists of 4 borosilicate tubes by which the reaction mixture was flown. Two aluminum parabolic mirrors concentrated the sunlight in the axis of each tube. The plant surface, tilted 30° with the horizon, was 0.26 m^2^, with an irradiated volume of 1.83 liters. The set-up was equipped with a radiometer (Acadus 85), which measured the received UV-A radiation. In each experiment, a reservoir was charged with 4 liters of the reaction mixture, which was continuously pumped into the plant, and then recirculated back to the reservoir. The samples were periodically taken from the reservoir for analysis.

In the experiment, real sunlight *t*_30*w*_ was used to normalize solar irradiation. It was calculated by Equation (1), where *UV_ac_* is the accumulated solar irradiation (J/m^2^), *V_i_*, and *V_T_*, are respectively, the irradiated and total volume (L), and I, the average light intensity (typically 30 W/m^2^ in Eastern Spain), as follows:

(1) $$t_{30w}=\frac{UV_{ac}\times V_{i}}{I\times V_{t}}.$$

### 2.3. Chemical Analyses

The concentration of PCP was followed by liquid chromatography. The apparatus employed was a LaChrom from Merck-Hitachi (Hitachi High-Tech Solutions Corporation, Fukuoka, Japan), equipped with auto-sampler and diode array detector (L-7459A). A reverse phase column LiChrospher^®^ 100 RP- was employed. A mixture of acetonitrile (A) and 0.001 M aqueous solution of sulphuric acid (B) in isocratic mode (80:20) was used as mobile phase, and the flow rate was 1 mL/min. Detection was based on the absorption at 220 nm and quantitation was performed by comparison with standards. The concentration of iron species was determined employing a spetrophotometric method based on ISO 6332:1998.

## 3. Results

### 3.1. Effect of Photolysis of PCP in Solar Simulator

**Figure 1:**
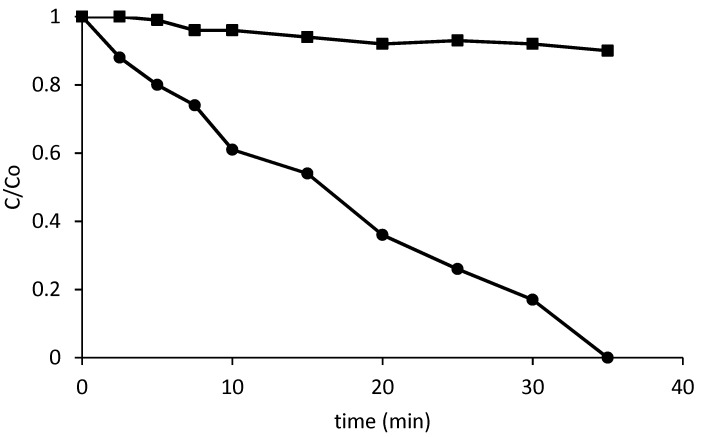
Plot of the relative concentration pentachlorophenol (PCP) decrease versus time when irradiated in a solar simulator without (●) and with (■) a Pyrex^®^ filter.

In a first series of experiments, the effect of the irradiation set-up was investigated. A solution of PCP (1 mg/L) was irradiated with a solar simulator with, and without, a pyrex glass filter able to cut off irradiation below 300. Although only a very small fraction of the photons were cut off by the filter, it represented the most energetic part and, as a result, very important differences can be appreciated in Figure 1: The PCP disappears quickly if the solution is directly exposed to solar light, whereas the presence of a Pyrex^®^ filter severely hinders the photolysis.

The photo-Fenton process was carried out at three different pH levels, with, and without, HLS in the absence of the Pyrex^®^ filter. The concentration of iron was 1 mg/L and the amount of hydrogen peroxide was the stoichiometric amount required to oxidize completely the PCP present in solution. Table 1 shows the time (min) required to reach 50% removal of PCP under the different conditions (t_50%_). It can be observed that the reaction was faster at pH = 2.8 (with a t_50%_ of 2 min), but efficient at pH = 5 (t_50%_ = 4 min). On the contrary, the lowest reaction rate was observed at pH = 7; in fact, at this pH, the reaction profile matches with photolysis (Figure 1) with a t_50%_ of 16 min in both cases, what means that PCP removal can only be attributed to photolysis. This behavior is in agreement with the pH dependence of photo-Fenton, which becomes less efficient when approaching to neutral pH.

Regarding the role of HLS, in all cases, its presence resulted in a higher t_50%_ and, consequently, in a loss of efficiency of photo-Fenton. This might indicate that the decrease in the direct photolysis of PCP attributable to the screen effect, on HLS or the scavenging role of these organic macromolecules, cannot be compensated by a better performance of photo-Fenton.

Once the effect of HLS on direct photolysis of PCP is determined, all the other experiments were carried out with the Pyrex filter. First, the same series of experiments, discussed above, were repeated (data not shown). Trends were similar, but in this case, the reaction rates were systematically lower, in particular, at pH = 7, where less than 20% removal was reached after 40 min of treatment. The detrimental role of HLS could also be observed, although it was not so remarkable as in the experiments driven without a filter. As the photolysis of PCP is negligible under these conditions, the decrease in the reaction rate could only be attributed to a shadowing of the HLS that did not allow an efficient photo-Fenton in the inner parts of the reactor or, more likely, to a competition of HLS for the reactive species.

### 3.2. Overcoming the Scavenging Role of HLS

Increasing the concentration of H_2_O_2_ might be a way of overcoming the competition between HLS and the pollutant for the reactive species. To check this point, the experiments were carried out twice and four times the amount of H_2_O_2_, with, and without, HLS at pH = 5. Table 2 shows that a net improvement in PCP degradation, in the presence of HLS, occurred, as shown by the t_50%_ values. This could be explained by considering that, at higher H_2_O_2_ concentration, the ·OH radicals production is enhanced, and it is reasonable to assume that the HLS sink effect towards ·OH radicals is compensated by the ability of this substance to form a photoactive iron complex, able to drive photo-Fenton-like reactions [11]. Actually the constant of this type of HLS with Fe(III) have been recently determined, showing their highest values at pH = 5 [10]. In other words, the addition of HLS can only be advantageous to photo-Fenton if there is enough peroxide, which represents the need to add extra amounts of this reagent, as already observed for the removal of thiabendazol at high concentrations [19].

### 3.3. Solar Pilot Plant Experiments and Role of Iron

The results obtained in the solar simulator cannot be extrapolated directly to the pilot plant. As a matter of fact, until this moment, very few data are available on the use of macro-molecules as complexing agents in photo-Fenton processes in a pilot plant [17]. Several experiments were carried out with the three studied pH levels and under two different conditions: (a) The stoichiometric amount of H_2_O_2_ without HL, and (b) 5 times the stoichiometric amount of H_2_O_2_ and HLS (5 mg/L). The addition of extra amounts of hydrogen peroxide in the experiments with HLS was done in order to avoid the competitive effect of this species towards PCP, as discussed in the previous section. The results in Table 3 show that HLS was not able to significantly enhance photo-Fenton, as only at pH = 2.8 a slight improvement could be observed. A very important photolysis of PCP can be observed in pilot plant experiments (t_50%_ = 11 min in the photolysis of PCP with, and without, H_2_O_2_) relevantly contributing to PCP degradation, rather than to photo-Fenton.

**Figure 2:**
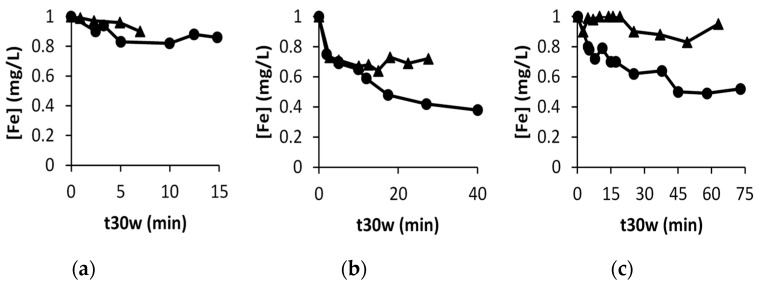
Evolution of Iron concentration in the absence (●) and in the presence (▲) of 5 mg/L of HLS: (**a**) at pH 2.8, (**b**) at pH 5 and (**c**) at pH 7.

Table 3 shows that the scavenging role of HLS and its shadowing effect compensate for the additional reactive species generated. In order to explain the poor performance of the Fe-HLS system, even when there is enough peroxide, the amount of iron present in active forms might be crucial, as its initial concentration was 1 mg/L, which is close to the amount that can be kept in solution even at a neutral value; as a matter of fact, using low iron concentrations is a strategy employed to drive photo-Fenton at neutral media [3]. In order to clarify this point, the concentration of iron present in solution was monitored according to the o-phenanthroline standard method [19], and results can be found in Figure 2.

A concentration of iron of ca. 0.5 mg/L was maintained, even after 75 min of normalized irradiation time even at pH = 7. This value is not far from the initial 1 mg/L that was added at the beginning of the process, and can explain why HLS are not able to enhance efficiently the generation of reactive species.

**Figure 3:**
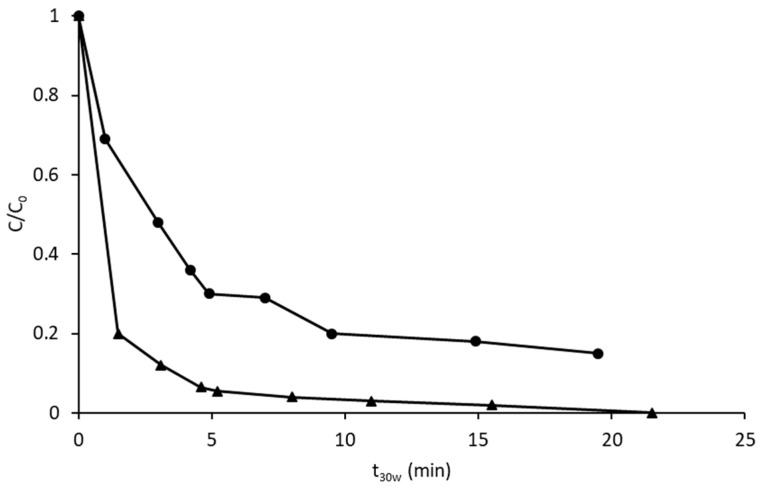
Degradation of PCP by photo-Fenton at pH 5 with the stoichiometric amount of H_2_O_2_, 4 mg/L of Fe(II) and in the absence (●) and in the presence (**▲**) of 20 mg/L of HLS.

In order to test this point, the experiments were repeated with 4 g/L of iron. They were carried out at pH = 5, where the effect of the HLS has been demonstrated to be efficient in previous papers, as indicated in the introduction. Figure 3 shows the reaction profiles with, and without, HLS. When HLS was present (20 mg/L), the amount of H_2_O_2_ was five times the stoichiometric amount, in order to compensate for the competitive role of HLS. Under these conditions, the positive role of HLS became more evident, as nearly all the PCP was removed after only 5 min of normalized irradiation, while ca. 20% of the initial amount of PCP remained in the solution at t_30W_ = 15 min, when HLS was absent. Iron was also monitored, and it was observed that for the HLS experiment, the iron concentration was systematically above 4 mg/L, while a remarkable decrease was observed in the absence of the complexing agent, reaching values below 2 mg/L at the end of the experiment.

## 4. Conclusions

The present work demonstrates the role of HLS as complexing agents, which drive the photo-Fenton process in mild conditions, and their function can be limited by factors, such as the type of substrate and its concentration. For those substrates that undergo strong photolysis, such as PCP, the inner filter effect of the HLS [20,21], as well as the possible filter effects, because of the reaction set-up playing a predominant role, thereby decreasing the efficiency of the process. In addition, under those conditions, the irradiation source is very relevant, as small changes in the irradiation spectrum can induce important variations in the rate of photolysis. Hence, experiments under real solar irradiation are necessary in these cases.

The pollutant concentration is also relevant, as low PCP to HLS ratios makes it necessary to increase the amount of hydrogen peroxide well above the stoichiometric amount required to oxidize the pollutant. In addition to this, the amount of iron cannot be decreased as a severe loss of efficiency in the process is observed.

Taking this into account, it is important to show the performance of HLS as an auxiliary for larger-scale, direct solar light photo-remediation of a wider number of pollutants, focusing on pollutants that undergo scarce photolysis, and when they are at relatively high concentrations. This would allow the assessment of the actual viability of HLS-assisted photo-Fenton application to real polluted waters remediation processes, and to promote the production and use of eco-friendly waste-derived chemicals to be used in place of fossil-derived products, in order to drive photo-Fenton at mild conditions [7,8,9,12].

## Figures and Tables

**Table 1:** Removal of pentachlorophenol (PCP) (1 mg/L) by photo-Fenton driven under simulated sunlight without Pyrex filter at different pH with or without HLS. The t_50%_ are given in min.

	pH = 2.8	pH = 5	pH = 7
0 g/L HLS	2	4	16
1 g/L HLS	5	7	16
5 g/L HLS	6	7	20
10 g/L HLS	12	30	More than 60

**Table 2:** Removal of PCP (1 mg/L) by photo-Fenton driven under simulated sunlight with Pyrex filter with, and without, HLS and with extra amount of hydrogen peroxide. The t_50%_ are given in min.

	with HLS	without
stoichiometric H_2_O_2_ × 2	10	17
stoichiometric H_2_O_2_ × 4	4	37

**Table 3:** Degradation of PCP by photo-Fenton at three different pH (2.8, 5 and 7) with the stoichiometric amount of H_2_O_2_ and five times this amount. PCP = 1 mg/L, Fe(II) = 1 mg/L, HLS = 5 mg/L. The t_50%_ are given in min. Photolysis was also measured in the absence H_2_O_2_ and presence of five time the stoichiometric amount of this reagent_2_, t_50%_ was 11 min for both cases.

	pH = 2.8	pH = 5	pH = 7
Stoichiometric H_2_O_2_	2	1.1	14
Stoichiometric H_2_O_2_ × 5 and HLS	1.1	0.9	14
